# Genetic determinants of Biofilm formation of *Helicobacter pylori* using whole-genome sequencing

**DOI:** 10.1186/s12866-023-02889-8

**Published:** 2023-06-01

**Authors:** Kartika Afrida Fauzia, Hafeza Aftab, Muhammad Miftahussurur, Langgeng Agung Waskito, Vo Phuoc Tuan, Ricky Indra Alfaray, Takashi Matsumoto, Michiyuki Yurugi, Phawinee Subsomwong, Evariste Tshibangu Kabamba, Junko Akada, Yoshio Yamaoka

**Affiliations:** 1 Department of Environmental and Preventive Medicine, Oita University Faculty of Medicine, Yufu, 879-5593 Japan; 2 Department of Public Health and Preventive Medicine, Faculty of Medicine, Universitas Airlangga, Surabaya, 60115 Indonesia; 3 Helicobacter pylori and Microbiota Study Group, Institute of Tropical Disease, Universitas Airlangga, Surabaya, 60115 Indonesia; 4 Department of Gastroenterology, Dhaka Medical College and Hospital, Dhaka, 1000 Bangladesh; 5 Division of Gastroentero-Hepatology, Department of Internal Medicine, Faculty of Medicine-Dr. Soetomo Teaching Hospital, Universitas Airlangga, Surabaya, 60115 Indonesia; 6 Department of Physiology and Biochemistry, Faculty of Medicine, Universitas Airlangga, Surabaya, 60115 Indonesia; 7 Department of Endoscopy, Cho Ray Hospital, Ho Chi Minh, 749000 Vietnam; 8 Department of Microbiology and Immunology, Hirosaki University Graduate School of Medicine, Hirosaki, 036-8562 Aomori Japan; 9 Research Center for Infectious Sciences, Department of Parasitology, Graduate School of Medicine, Osaka City University, Osaka, Japan; 10 Department of Medicine, Gastroenterology and Hepatology Section, Baylor College of Medicine, Houston, TX 77030 USA; 11 Borneo Medical and Health Research Centre, University Malaysia Sabah, Kota Kinabalu, Sabah, 88400 Malaysia; 12 The Research Center for GLOBAL and LOCAL Infectious Diseases (RCGLID), Oita University, Yufu, 879-5593 Oita Japan

**Keywords:** Whole-genome sequences, SNP, Variants, Biofilm formation, Antibiotic resistance, Infectious disease

## Abstract

**Background:**

Infection with *Helicobacter pylori* as the cause of gastric cancer is a global public health concern. In addition to protecting germs from antibiotics, biofilms reduce the efficacy of *H. pylori* eradication therapy. The nucleotide polymorphisms (SNPs) related with the biofilm forming phenotype of *Helicobacter pylori* were studied.

**Results:**

Fifty-six *H. pylori* isolate from Bangladeshi patients were included in this cross-sectional study. Crystal violet assay was used to quantify biofilm amount, and the strains were classified into high- and low-biofilm formers As a result, strains were classified as 19.6% high- and 81.4% low-biofilm formers. These phenotypes were not related to specific clades in the phylogenetic analysis. The accessories genes associated with biofilm from whole-genome sequences were extracted and analysed, and SNPs among the previously reported biofilm-related genes were analysed. Biofilm formation was significantly associated with SNPs of *alpA, alpB, cagE, cgt, csd4, csd5, futB, gluP, homD*, and *murF* (P < 0.05). Among the SNPs reported in *alpB*, strains encoding the N156K, G160S, and A223V mutations were high-biofilm formers.

**Conclusions:**

This study revealed the potential role of SNPs in biofilm formation and proposed a method to detect mutation in biofilm from whole-genome sequences.

**Supplementary Information:**

The online version contains supplementary material available at 10.1186/s12866-023-02889-8.

## Introduction

*Helicobacter pylori* infection remains a public health problem worldwide, affecting half of the humans and inducing various gastrointestinal tract diseases [1]. The acidic and hostile conditions of the human stomach constitute the natural niche of *H. pylori*, suggesting high adaptation abilities [2, 3]. Biofilm-forming *H. pylori* strains have been observed in vivo on the surface of the gastric mucosa [4]. The biofilm is a complex exopolysaccharide structure that protects and maintains life in the presence of external stress [5]. The biofilm also protects bacteria against antibiotics, resulting in a decline in *H. pylori* eradication [6, 7]. Previous studies reported that high-biofilm formers are likely more resistant to antibiotic exposure [7, 8]. Therefore, the prediction of genetic determinants for biofilm formation is necessary. Understanding the mechanism of biofilm formation is essential for improving *H. pylori* elimination strategies.

Approaches that enable the investigation of genetic mechanisms correlated to a given phenotype are currently available. In the first approach, the gene responsible for a phenotype is studied by generating a mutant of the target gene from the wild-type strain and observing the phenotype [9]. For example, removing the entire gene of *alpB* was found to inhibit the adhesion and initiation of biofilm formation [10, 11]. In the second approach, the genetic variation that existed in the natural population was evaluated [9, 12]. Statistical analysis is performed on the dataset of strains with two distinctive traits. Genetic variants associated with traits are candidates for explaining mechanisms of the trait seen in the population. This approach has advanced quickly, particularly with the development of whole-genome sequencing methods. Previous studies assessed the SNPs in *gyrA, gyrB*, and *23s rRNA* that is involved in AMR (Antimicrobial Resistance) in order to decipher the mechanism of AMR. [13] [14]. This similar approach is potentially applied to the other phenotype, such as the bacteria’s ability to form biofilm. Genetic variation, such as insertion, deletion, and SNPs in the high and low biofilm, is a field that deserves further investigation. Therefore, using a similar approach as in antibiotic resistance, this study investigates the potential use of the genomic approach to evaluate biofilm formation.

Biofilm formation levels among the strains varied from low to high, indicating a potential complex mechanism involving a specific genotype or variant [6, 15]. The level of genetic variation can be the present absence of genes or the SNP. SNPs that consist of insertion, deletion, and non-synonymous point mutation could be one possible mechanism for biofilm formation [16]—identifying the point mutation and which genes for the focused evaluation have been challenging. Various tools have been developed to assess single nucleotide variants (SNVs), such as “Antimicrobial Resistance Identification By Assembly” (ARIBA), that can be used to assemble targeted genes and detect the presence or absence of genes and nucleotide variants [17]. Meanwhile, finding new candidate genes among the genomes at the present-absent level is also necessary [18, 19].

We performed the genotype-phenotype evaluation of biofilm on the *Helicobacter pylori* clinical isolates obtained from Bengali ethnic Bangladesh subjects. We investigated the novel nucleotide variant of a list of genes reported to be involved in biofilm formation as a starting point. The steps included confirming those genes’ presence in the clinical isolates and discovering the nucleotide alteration variant that may involve biofilm phenotype shift. We also assessed the other possible novel genes associated with biofilm phenotypic alterations [18].

## Results

### Distribution of biofilm formation among *H. pylori* strains from Bangladesh

**Fig. 1:**
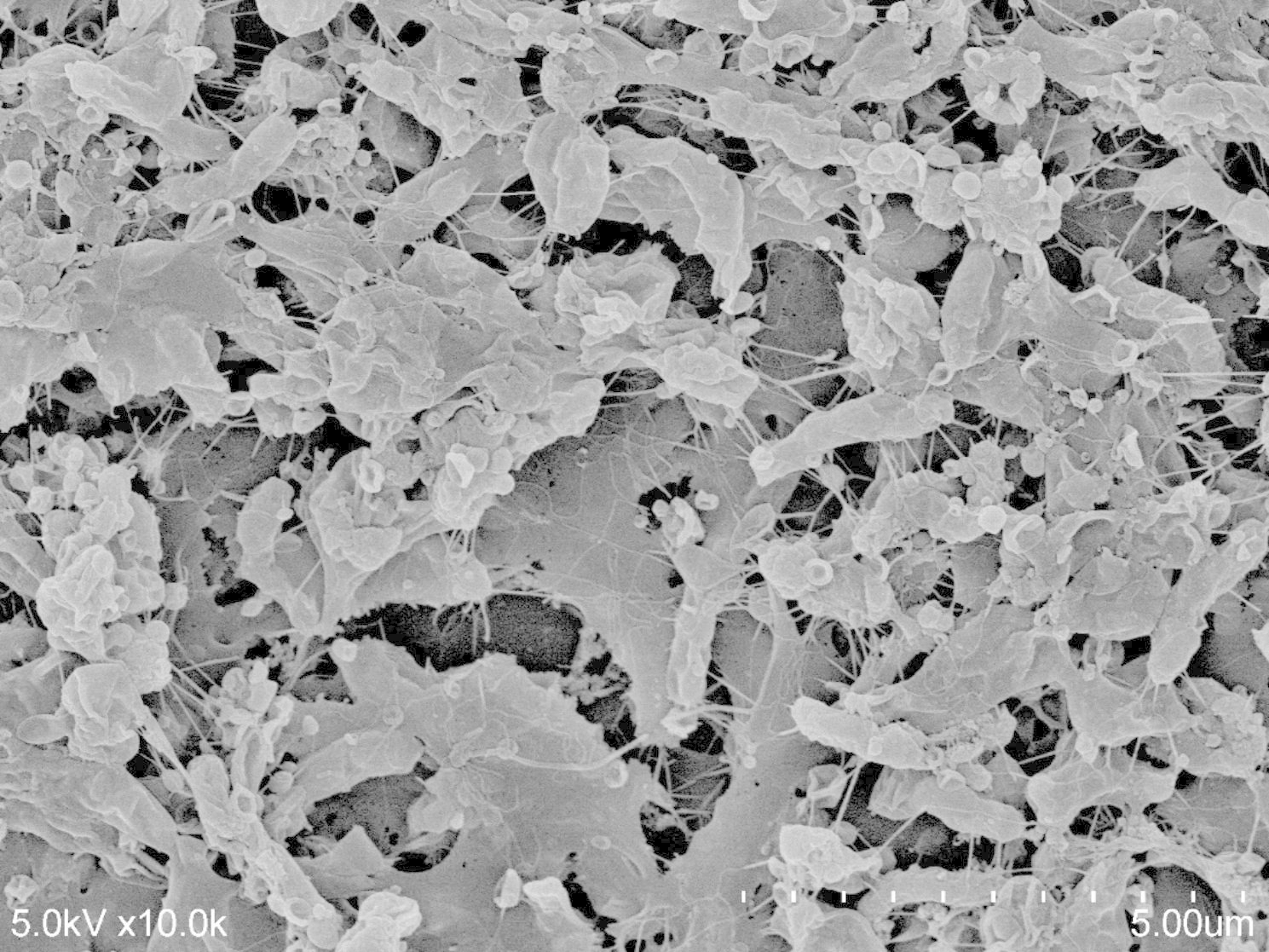
The morphology of biofilm formation on the coverslip surface on the day 4 of culture can be seen in this figure (10,000X magnification). The rod-shaped cells were covered with thick structure

Biofilm formation was quantified and classified into two groups: high- and low-biofilm formers (Supplementary Figure S1) were determined using crystal violet assay. The group of high-biofilm formers included 19.6% of strains (11/56) with a mean optical density (OD) of 0.85 (SD 0.4). The distribution of low-biofilm formers was 80.4% (45/56) and a mean OD of 0.24 (SD 0.06). Among the low-biofilm former, 11 strains of the low-biofilm formers had OD below control. One representative high biofilm strain (BGD114) confirmed its dense biofilm formation by observation using the SEM (Fig. 1). The growth curve pattern of the isolates was comparable; therefore, the biofilm formation OD was independent of growth (Supplementary Figures S2a and S2b).

### The phylogenetic similarity among the biofilm formers

**Fig. 2:**
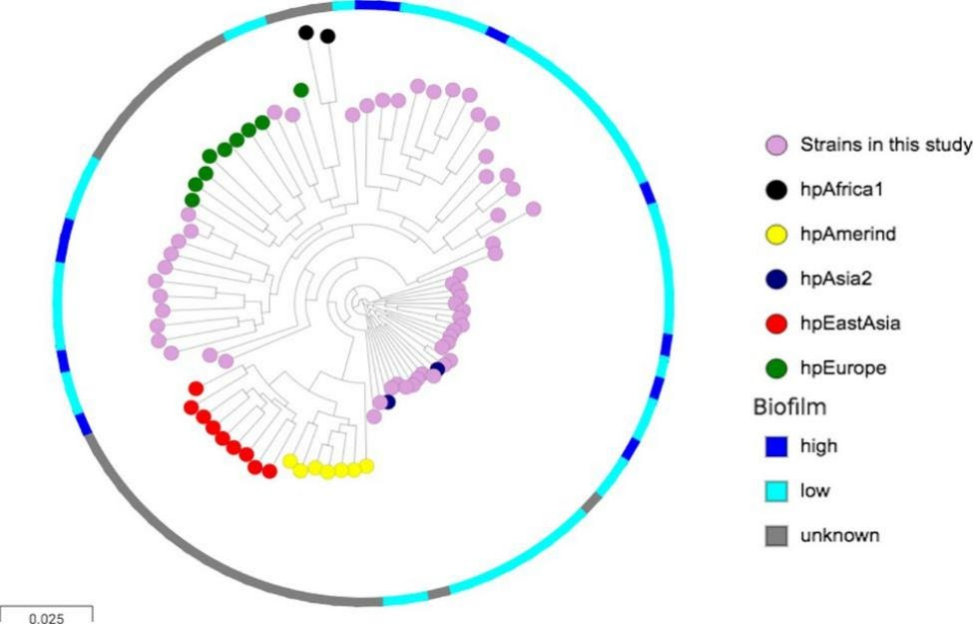
The phylogenetic tree depicts the population and the biofilm formation The circle represents the population’s genetic type. The big circle with dark blue and light blue represented the high and low biofilm phenotype, respectively (grey: unknown because biofilm test was unavailable)

A phylogenetic tree inferred by the maximum likelihood algorithm was used to assess the eventual association between the biofilm formation phenotype and different genetic *H. pylori* lineages (Fig. 2). High biofilm was sporadically distributed among the clades; thus, no specific phylogenetic branch was associated with biofilm formation. In the genomic population of *H. pylori*, the strains were assigned using published reference strains [20]. The *H. pylori* genomic population has been previously described as hpAfrica1, hpAmerind, hpAsia2, hpEastAsia, and hpEurope. Using the phylogeny approach, the strains in this dataset belong to hpAsia2 and hpEurope. No significant genomic-population association was found between the genome population type and biofilm formation. Among the hpEurope strains, 21.6% (3/19) of the high biofilm formation was observed, and only 15.8% (8/37) was observed in hpAsia2.

### The association of the presence and absence of well-known genes to biofilm formation

We compiled the research findings of studies investigating the genes which play roles in biofilm formation. Then we summarized up to 46 genes that will be used as targets for analysis of the Bangladeshi genomes in this dataset (Supplementary Table S2). Using the minimum coverage criteria of 50% and a minimum identity of 90%, 32 of the 46 genes were present in all isolates (Supplementary Table S2). The genes absent in more than 10% of the isolates were *vapD, cagD, cagE*, and *csd5*. The Fisher exact test did not give a significant P-value. This result indicated that the absence of the genes above rarely occurred naturally and may not be the only factor contributing to biofilm formation.

### The variants associated with biofilm formation

The SNPs were analyzed to find out the mechanism related to biofilm formation (other than the gene absent). This pipeline assembles the gene according to the sequences in the references, ATCC 26695, which has low biofilm formation. The genes investigated in this study played a role in biofilm formation. The total number of polymorphisms extracted from the analysis with the MAF > 10% was 960, including frameshift, insertion, deletion, multiple site polymorphism, and single nucleotide polymorphism, summarised in (Fig. 2). Comparison of the variant found between the high and low biofilm former. A total number of SNPs were observed among the investigated genes, the highest number of SNPs was observed in the *homB* gene (279 variant), and the lowest was observed in *cheY* genes (1 variant). The statistically significant variants for the biofilm phenotype are listed in Table 1. The SNPs in adhesion-related genes were included, such as *alpA, alpB, homD, cagE*, and *futB*. Other genes that have roles in metabolism and cell shape regulation, such as *gluP, cgt, csd4, csd5, murF*, and *amiA*, also showed significant associations. Three SNPs in the *alpB* gene were significantly associated with the high-biofilm former at A223V, G160S, and N156K. The gene expression of the *alpB* gene was also higher in the high biofilm former compared to low biofilm former isolates (Supplementary Figure S3).

**Table 1:** The significant variants associated with biofilm formation

Gene	SNP	Proportion in Low Biofilm	Proportion in High Biofilm	Naïve	FDR	Reference
				P-value		
*alpA*	G196N	0.16	0.45	0.045	0.045	[11]
*alpB*	A223V	0.31	0.73	0.017	0.045	[11]
	G160S	0.38	0.91	0.002	0.036	
	N156K	0.09	0.45	0.01	0.045	
*amiA*	T168V	0.07	0.36	0.022	0.045	[21]
*cagE*	S419C	0.42	0.87	0.045	0.045	[15]
*cgt*	V34A	0.178	0.54	0.02	0.045	[22]
*csd5*	M125I	0.15	0.56	0.017	0.045	[21]
	P43S	0.1	0.44	0.026	0.045	
	V110A	0.07	0.44	0.015	0.045	
*futB*	E98fs	0.16	0.5	0.033	0.045	[15]
*gluP*	T85S	0.27	0.64	0.032	0.045	[23]
*homD*	A570T	0.09	0.36	0.04	0.045	[15]
	V249fs	0.13	0.45	0.029	0.045	
*murF*	V250I	0.33	0	0.026	0.045	[21]
**Gene**	**SNP**	**Proportion in Low Biofilm**	**Proportion in High Biofilm**	**P-value**	**FDR**	**Reference**
*alpB*	T127A	0.911	0.636	0.04	0.045	[11]
*csd4*	C12Y	0.711	0.364	0.041	0.045	[21]
*futB*	L317I	0.432	0	0.01	0.045	[15]

**Fig. 3:**
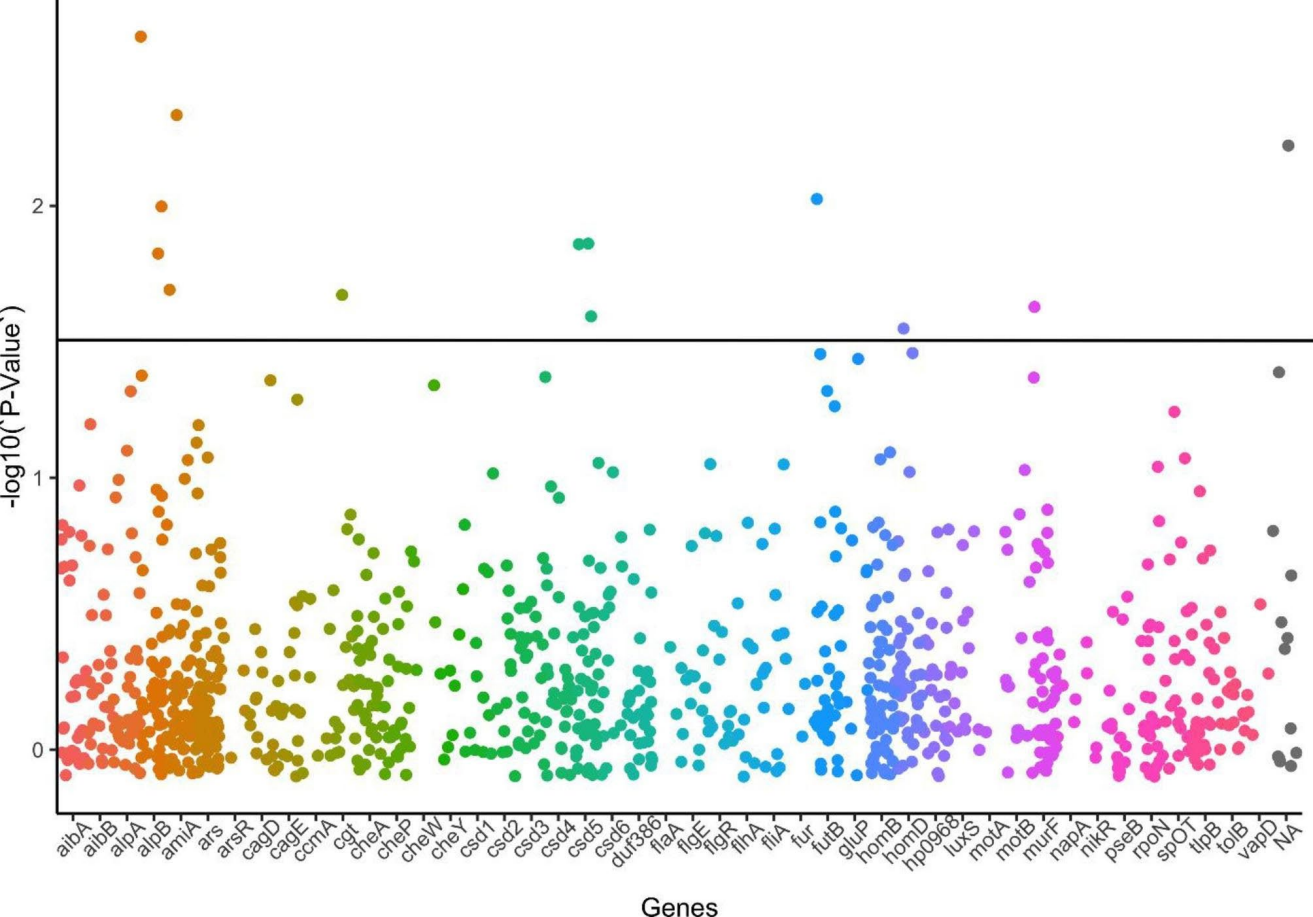
Scatter plot of P-values of the nucleotide polymorphisms (SNPs) in the target genes and their association with biofilm formation

The P-values observed in all nucleotide polymorphisms (SNPs) of all genes are summarised in Fig. 3. The x-axis represents the lists of SNPs, and the y-axis represents the p-value from fisher exact tests for the genes and the biofilm phenotype. The straight horizontal line represents the cut-off for the significant P-value (0.05). The dots show the P-value of each variant, and 18 dots above the line have a P-value less than 0.05.

### The presence of mutation and association to phenotype in the independent dataset

To evaluate whether the mutation is also present in other strains in the population, we randomly selected 20 strains of *H. pylori* clinical isolates from Indonesia as the validation dataset from the previous study [24]. This dataset comprised the high biofilm former (n = 7) and low biofilm former (n = 13). Each isolate’s genes subjected to the validation (*alpB, cgt,gluP, csd4, csd5, murF*) were extracted and aligned to 26695. This dataset assessed the presence of mutation among the low and high biofilm former (Supplementary Table S3). Even if these minor number validation data sets, substitution in the *cgt* genes V34A occurred only in the high biofilm former. A similar tendency was observed in the strains possessing A223V and N156K substitutions in *alpB* genes. Each SNP and biofilm formation OD was plotted (Supplementary Figure S4). The variation of mutation proportion between high and low biofilm formation was observed between the original and validation dataset and the average biofilm formation among the SNP.

### Screening of accessories genes associated with biofilm formation

The whole-genome sequences were annotated, and the pan-genome was constructed. Then the genotype-phenotype association analysis was performed on the accessories genes using a scoary pipeline. The genes naturally absent in certain strains are called accessory genes and can contribute to phenotypic change. The genes presented in < 99% of pan-genome with a minimum identity of 60 and coverage of 50 were used to determine accessory genes. The analysis discovered that 28% of the DUF1524 was absent in the high biofilm former [25]. The results also revealed several genes (Table 2) that functioned in DNA replication (DNA polymerase, DNA primase). Five genes related to gene insertion, such as phages (*dB-ParB* domain-containing protein of phage, Helicobacter phage *FrB41M*, putative tail assembly protein of phage), insertion sequence (IS, *IS200/IS605 family transposase IS606*,) and plasticity gene (*VirB4 homolog*) were discovered.

**Table 2:** Accessories gene associated with biofilm formation

Gene	Annotation	Present in low biofilm (n)	Present in high biofilm (n)	Proportion of gene present in biofilm level		Naïve P value	FDR
				High	Low		
btuB_1	Vitamin B12 transporter BtuB	10	24	0.909	0.533	0.036	1
group_1745	Phosphoethanolamine transferase CptA	9	21	0.818	0.467	0.047	1
group_485	DUF1524 domain-containing protein	8	45	0.727	1	0.006	1
group_824	G domain-containing protein	8	45	0.727	1	0.006	1
group_243	hypothetical protein	8	16	0.727	0.356	0.041	1
group_2979	VirB4 homolog (VirB4)	4	4	0.364	0.89	0.04	1
group_2579	DNA primase	3	2	0.273	0.44	0.047	1
group_2913	IS200/IS605 family transposase IS606	3	1	0.273	0.22	0.021	1
group_3500	ddrB-ParB domain-containing protein of phage	3	1	0.273	0.22	0.021	1
group_3675	Putative tail assembly protein of phage	3	2	0.273	0.44	0.047	1
group_363	hypothetical protein	3	2	0.273	0.44	0.047	1
dnaE2_1	Error-prone DNA polymerase	2	0	0.182	0	0.036	1
group_4028	Uncharacterized protein (Helicobacter phage FrB41M)	2	0	0.182	0	0.036	1
group_2993	hypothetical protein	2	0	0.182	0	0.036	1
group_290	conserved hypothetical ATP-binding protein	0	16	0	0.356	0.023	1

## Discussion

Biofilm formation is a beneficial mechanism that requires complex regulation and interaction between bacteria. In *H. pylori*, in vitro observations of mono-species biofilms showed that high biomass was obtained after 72 h [21, 26]. The evaluation of strains under the same conditions showed that biofilm formation was significantly higher in particular strains, indicating a variation in biofilm formation. These variations were also observed in the present study. Among the strains from the Bangladesh dataset, 19.6% of the strains could be categorized as high-biofilm formers.

In some bacterial species, biofilm formation can be associated with specific lineages or population, e.g., in *Staphylococcus aureus* [27]. However, this association in *H. pylori* remains questionable. Therefore, we tried to adress this question by constructing a phylogenetic tree of Bangladeshi isolates using the SNPs-based core genome alignment tree. The result suggested that there was no association of biofilm formation to a specific lineage in *H. pylori*. This result was supported by other study reported that there is no association between specific lineages or population with biofilm formation [28].

The whole-genome sequence analysis allow researchers to investigate genes and mutations related to biofilm formation. Hence, we evaluated the presence and absence of genes potentially related to biofilm formation (adhesion, shape formation, efflux pump, and even dispersion, as listed in Supplementary Table 2) then obsereved their difference between high-and low biofilm formation group in the Bangladeshi isolates. Our results showed that these genes were present in most isolates, despite variations in the biofilm formation. This condition makes identificatation of the gene marker for the high-biofilm formers in *H. pylori* remains challenging. While previous study showed that the presence status of several genes such as *cagD, futA*, and *napA*, could be associated with the biofilm level [25], this result was not present in this study. Nevertheless, further study should be done to identify polymorphism of the targeted genes that may affect the phenotype.

Next, we focused on amino acid variants analysis, including insertion, deletion, missense mutation, or SNPs. This study is the first to assess the SNPs associated with biofilm formation in *H. pylori.* Several SNPs have been linked to specific phenotypes of *H. pylori*, such as diseases like gastric cancer [16]. SNPs or missense mutations are also associated with resistance to clarithromycin, levofloxacin, and rifampicin [29]. The mutation in the targeted gene, such as *23 S rRNA*, *gyrA*, and *gyrB*, was concordant with antibiotic resistance, and the statistical analysis showed significant results. Therefore, the identification of SNP on the target genes could be informative. We observed that the gene with highest variants is *homB.* Based on the molecular studies that have been conducted, HomB is an outer membrane appears to be involved in adherence [30]. Outer membrane protein has high variant as a mechanism to adapt to the various host. Although this has high variant, no significant hits were found in this dataset. Meanwhile, the lowest number of variant were present in *cheY* gene that regulates the direction of flagella motor Due to its critical role in survival, motility related gene has been conserved among archae and bacterial [31]. It has conserved active site residues which are activated by phosphorylation mechanism [32]. The flagella regulation play roles in switching the planktonic and biofilm phenotype. However, the significant variant was not found in this dataset.

We constructed a database of the genes from previous studies associated with biofilm formation to analyze the dataset from Bangladesh. As a result, 11 genes with variants significantly related to biofilm formation were identified. Significant nucleotide polymorphism were found in the genes that are involved in the cell shape regulation ( *gluP, cgt, csd4, csd5, murF, and amiA*) [33]. These genes regulate the change of helical form to coccoid forms which are commonly observed in biofilm of *H. pylori* [34, 35]. This finding implies that the cell-shape regulation deserves a spotlight in the biofilm formation mechanism study.

Virulence gene such as *cagE* and *cagD* that belong type IV secretion system (T4SS) were also detected. This T4SS secreted CagA and other products were proposed to interplay with *luxS ;* the quorum sensing gene. As reported in the previous study, the genetic modification on *cagE* showed a significant alteration of biofilm formation [25, 36, 37]. Furthermore, variants in five genes encode outer membrane proteins (OMPs) of aherewere also discovered [38–40]. The variants in *alpA and alpB* were significantly associated with the high biofilm formation. Those genes encode OMPs which indicate that adherence and aggregation process is necessary for biofilm formation. The significant variant that we found were adjacent to the variable region of *alpB* that previously reported to play role in the biofilm formation (locus 121–150) [38]. Therefore, to check the possibility of the gene expression change related to the present of mutation, we performed RT-PCR for the *alpB* gene. The higher expression of gene were presented by the high biofilm former strains, indicate the increased activity. Because multiple factors could influence the gene expression, mutagenesis on the fragment containing polymorphism is necessary to confirm the result.

After finding the SNPs, validation is essential [41]. For this purpose, we used the data set of *H. pylori* clinical isolates obtained from Indonesia. A coherent result could be observed in the *cgt* and *alpB*, indicating a possible biomarker for biofilm formation. The results also implicate SNP’s contribution in those genes as part of many factors involved in building biofilm phenotype. Nevertheless, we should be cautious with the possible false-positive results, shown by the inconsistent result in other SNPs compared to the primary dataset (Supplementary Figure S3).

The first step of the analysis only targetting the well-known mutation. Thus, we broaden the analysis of all genes throughout the genome. The gene-to-phenotype association study of the accessories gene by the Scoary pipeline discovered a gene annotated *DUF1524* associated with the high biofilm formation reported in the previous study [25]. The function of *DUF1524* and its roles in biofilm formation is unclear and thus requires further experimental study. The results also found genes related to a mobile genetic element that could be related to the evidence that the biofilm environment enhances the mutation and genetic exchange [42–44].

The main limitation of the genotype-phenotype association of bacteria isolates is the number of samples that could be tested, the high recombination, and geography-specific adaptation [45]. The small number of samples included in this dataset and the statistical approaches applied cannot avoid the possibility of false-positive discovery among the detected SNPs. However, it could be a stepping stone for further molecular studies elucidating the genetic factors involved in biofilm formation and related molecular mechanisms.

## Conclusions

The association analysis of the SNPs in the well-known biofilm formation genes proved the ability to screen biofilm-formation capacity from whole-genome sequencing data. We observed a significant association between the SNPs, including *alpA, alpB*, *cagE*, and *csd4*. The next-generation sequence evaluation could be a method to decipher the mechanism of biofilm formation.

## Methods

### Patient sampling and *H. pylori* isolates

The isolates used as the primary dataset in this study were obtained from gastric biopsies of patients from a survey involving 133 subjects from Dhaka Medical College Hospital in 2014 [46]. To obtain a single colony of *H. pylori* isolates, biopsy specimens from the antrum were homogenized in phosphate-buffered saline (PBS). The biopsy was inoculated in *H. pylori* selective plates and incubated for five days in a 37 °C microaerophilic environment. Each colony was then subcultured in Brucella agar supplemented with 7% horse blood before harvesting for genomic DNA extraction. *H. pylori* obtained 56 isolates from patients with chronic gastritis (53/56) and peptic ulcers (3/56). The Oita University Faculty of Medicine Japan and the Ethics Committee of the Bangladesh Medical Research Council (BMRC), Dhaka, Bangladesh, approved the protocol of this study.

### Biofilm quantification

Biofilm quantification was performed using the crystal violet method for *H. pylori* with modifications already described in the previous study [44]. Briefly, the blood plate-grown bacteria were pre-cultured for 24 h under microaerophilic conditions in 1 mL Brucella broth supplemented with 10% fetal bovine serum (FBS). Adjusting the bacterial suspension to an optical density of 0.4 (approximately 2.5 × 10^6^/µL), 25 µL of H. pylori suspension was added to 24-well plates containing 1 mL medium. Three days were spent incubating these plates in a microaerophilic environment with shaking (100 rpm). The suspension of planktonic cells was then discarded. The amount of biofilm was determined by measuring the absorbance at 595 nm with a spectrophotometer (Multiskan Go, Thermo Fischer, Japan). The measurement of the well-containing medium devoid of bacteria was used as a negative control. A low biofilm-former had an optical density (OD) 2X the OD of the control sample, whereas a high biofilm-former had an OD 2X the OD of the control sample [47, 48]. The average OD of the control group was 0.2, so 0.4 was determined to be the high biofilm former.

In conclusion, two phenotype groups were identified: low-biofilm formers (low and negative) and high-biofilm formers (high biofilm former strains). Due to the use of reference strain 26695 genes in constructing the reference database, the biofilm formation of reference strain 26695 was also examined but not included in the dataset. The OD of the 26695 strain biofilm was 0.35, less than 0.4, so it was also categorized as a low biofilm producer. Each experiment was conducted in triplicate.

From day 1 to day 4, the bacterial growth was also monitored by spectrophotometry. A 24-well plate is loaded with 1 ml of the same bacterial solution for biofilm quantification. This liquid culture was incubated at 37 degrees Celsius with 10% CO_2_ and 100 rpm shaking. Each day, the optical density was measured.

### Genome sequencing

Genome sequence data were used in a previous study [46]. According to the manufacturer’s instructions, the *H. pylori* were harvested in PBS, and the DNA was extracted using the Qiagen DNEeasy Kit (Hilden, Germany). The DNA concentration was then measured using the Quantifluor dsDNA System (Madison, USA) and Quantus Fluorometer (Sunnyvale, USA). After standardizing the concentration, whole-genome sequencing was performed using MiSeq Illumina to obtain paired-end reads with a 300 bp length. The quality assessment by QUAST was summarised in Supplementary Table S1.

To analyze the genetic relatedness and the influence of population genetics, we created the whole genome alignment of all Bangladesh sequences and sequences from a previous study that identified the population [20]. The alignment was performed using the Snippy-core (ver 4.6.2) (https://github.com/tseemann/snippy). The alignment was used to construct a maximum likelihood tree using FastTree 2.0, with GTR–nt mode [49]. The tree was then visualized using Microreact [50].

### Analysis of the SNP

The analysis of the SNP used in the study was performed using ARIBA pipeline[51]. First, 42 genes of strain 26695 that were related to biofilm formation mentioned in previous studies were collected to create a reference database (Table S2). The metadata for ARIBA was set into coding sequences and new variants. These references are clustered based on the CD-HIT. Subsequently, the raw reads (fastq format) of the 56 strains were mapped to the reference sequence in the cluster and assembled independently. The reads were mapped to the cluster references with Bowtie, and the variants were called with SAMtools. The results of each strain consisted of the assembled genes and the report of variants compared to strain 26695. The reports were then summarised in a text file. The genes were present if the percent coverage was more than 50% and the percent identity was more than 90%. Then, a summary of SNPs with amino acid changes was shown, and the SNPs presented in less than 10% of the strains were excluded. The presence and absence of SNP were calculated to determine the association with biofilm formation. A Fisher exact test result of P-value < 0.05 was considered significant. The other non-parametric correlation was analyzed using Spearman’s rank correlation model. All statistical analyses and graph construction were performed using R (version 3.5.1).

### RNA extraction and measurement of the Gene expression

*Helicobacter pylori* clinical isolates were randomly selected from high (BGD114, BGD112, and BGD104) and low biofilm former strains (BGD96, BGD109). The high biofilm former possessed the mutation in A223V, G160S and N156K in the *alpB* genes while the low biofilm former isolates does not have any mutations in the loci above. The gene expression levels of *alpb* and *ppa* were measured. RNA was isolated from *H. pylori* cultured for 36 h. Total RNA from the bacteria was isolated using a commercially available kit (PureLinkTM RNA Mini Kit; Invitrogen by Thermo Fisher Scientific Inc.). The total RNA was quantified using the QuantiFluor® RNA System (Promega). A total of 120 ng RNA was converted into cDNA by using the PrimeScript™ RT Master Mix (Perfect Real Time) with the final product volume of 10 µl (Takara Bio Inc.). Real-time PCR was performed using iTaq Universal SYBR Green Supermix (Bio-Rad Laboratories, Inc.). A standard curve was constructed with 5-fold serial dilutions for each gene target. The expression of the genes was analyzed by using absolute method quantification.

### Analysis of accessories gene

This study used a set of genes to analyze the SNPs. To investigate if the genomes’ remaining genes (especially accessories genes) could also be associated with biofilm formation, we created a whole genome assembly for each strain. The reads was trimmed (ver 0.32) by trimmomatic and *denovo -* assembled using Spades (ver 3.1) [52, 53]. The results annotated by Prokka (ver 1.4) and the gff files output are used to construct a pan-genome by Roary (ver 3.11) [54, 55]. The gene –presence-absence result from Roary and the metadata containing high-low biofilm is used as the input for the Scoary (ver 1.6.16 [18].

## Electronic supplementary material

Below is the link to the electronic supplementary material.

Supplementary Material 1

## Data Availability

All genome data were stored in Genbank with BioProject accession PRJDB11821 (Available at: https://www.ncbi.nlm.nih.gov/bioproject/?term=PRJDB11821).
