# Supplementary material for: Genetic determinants of Biofilm formation of Helicobacter pylori using whole-genome sequencing
Source: BMC Microbiol. 2023 Jun 1;23:159. doi: 10.1186/s12866-023-02889-8 (PMC10234030; doi:10.1186/s12866-023-02889-8)
Supplement: Supplementary file 1 — Supplementary Material 1 [file 12866_2023_2889_MOESM1_ESM.docx]

**Supplementary Table S1. The quality of *H. pylori genomes***

| **Assembly** | **# contigs** | **Largest contig** | **Total length** | **Reference length** | **GC (%)** | **Reference GC (%)** | **N50** | **NG50** | **N75** | **NG75** | **L50** | **LG50** | **L75** | **LG75** | **Average Length** | **Number of Reads** | **Coverage** |
| --- | --- | --- | --- | --- | --- | --- | --- | --- | --- | --- | --- | --- | --- | --- | --- | --- | --- |
| BD0003 | 123 | 128077 | 1716262 | 1667867 | 38.8 | 38.87 | 52661 | 52661 | 27747 | 29326 | 11 | 11 | 23 | 21 | 170.5 | 800000 | 79.47504519 |
| BD0010 | 109 | 244270 | 1682427 | 1667867 | 38.66 | 38.87 | 52682 | 52682 | 31935 | 32394 | 10 | 10 | 21 | 20 | 70 | 3400000 | 141.4623042 |
| BD0013 | 48 | 280740 | 1577856 | 1667867 | 39.08 | 38.87 | 94596 | 94596 | 45339 | 40878 | 6 | 6 | 12 | 13 | 175 | 800000 | 88.72799546 |
| BD0014 | 61 | 181374 | 1607002 | 1667867 | 39.04 | 38.87 | 66750 | 66750 | 43012 | 39266 | 8 | 8 | 15 | 16 | 171 | 600000 | 63.84559571 |
| BD0015 | 36 | 210724 | 1562121 | 1667867 | 39.18 | 38.87 | 95807 | 95614 | 56683 | 46097 | 6 | 7 | 11 | 13 | 188 | 500000 | 60.17459595 |
| BD0016 | 48 | 317789 | 1613713 | 1667867 | 39 | 38.87 | 82554 | 82554 | 52522 | 49217 | 6 | 6 | 12 | 13 | 151.5 | 700000 | 65.71800562 |
| BD0024 | 34 | 293450 | 1618178 | 1667867 | 38.83 | 38.87 | 108598 | 99671 | 68064 | 56466 | 4 | 5 | 9 | 10 | 171.5 | 600000 | 63.59003768 |
| BD0027 | 60 | 195858 | 1607626 | 1667867 | 38.94 | 38.87 | 88095 | 88095 | 37498 | 31714 | 7 | 7 | 14 | 16 | 169.5 | 600000 | 63.26098234 |
| BD0034 | 34 | 268157 | 1605489 | 1667867 | 38.97 | 38.87 | 124246 | 102922 | 78533 | 78533 | 4 | 5 | 9 | 9 | 146.5 | 1300000 | 118.6242945 |
| BD0036 | 56 | 149136 | 1646969 | 1667867 | 38.86 | 38.87 | 64678 | 58511 | 35718 | 35718 | 9 | 10 | 19 | 19 | 140 | 1600000 | 136.0074173 |
| BD0038 | 40 | 195847 | 1576976 | 1667867 | 39.14 | 38.87 | 83429 | 83429 | 52793 | 49092 | 7 | 7 | 12 | 13 | 168.5 | 700000 | 74.79505078 |
| BD0042 | 98 | 185295 | 1694043 | 1667867 | 38.84 | 38.87 | 81846 | 81846 | 47258 | 47258 | 8 | 8 | 15 | 15 | 147 | 1600000 | 138.8394509 |
| BD0043 | 58 | 199360 | 1654489 | 1667867 | 38.78 | 38.87 | 82394 | 82394 | 43541 | 43541 | 7 | 7 | 15 | 15 | 139 | 700000 | 58.80969895 |
| BD0045 | 50 | 232130 | 1589933 | 1667867 | 38.99 | 38.87 | 70616 | 63153 | 39672 | 38491 | 7 | 8 | 15 | 16 | 132 | 1100000 | 91.32460299 |
| BD0046 | 41 | 217476 | 1608852 | 1667867 | 39.09 | 38.87 | 130946 | 94199 | 64635 | 58730 | 5 | 6 | 10 | 11 | 148.5 | 1000000 | 92.30184007 |
| BD0047 | 55 | 135207 | 1669133 | 1667867 | 38.73 | 38.87 | 60814 | 60814 | 47572 | 47572 | 9 | 9 | 17 | 17 | 164 | 700000 | 68.77822199 |
| BD0049 | 121 | 124410 | 1759006 | 1667867 | 38.68 | 38.87 | 62199 | 68597 | 26556 | 33256 | 10 | 9 | 21 | 18 | 163 | 800000 | 74.13277726 |
| BD0052 | 42 | 246164 | 1591320 | 1667867 | 39.07 | 38.87 | 66714 | 66714 | 41418 | 39413 | 6 | 6 | 13 | 15 | 144 | 2300000 | 208.1291004 |
| BD0053 | 432 | 62565 | 2030725 | 1667867 | 38.73 | 38.87 | 13567 | 16694 | 4249 | 8503 | 41 | 29 | 106 | 62 | 146 | 1100000 | 79.08505583 |
| BD0054 | 61 | 172458 | 1662617 | 1667867 | 38.79 | 38.87 | 59497 | 59497 | 38131 | 38131 | 9 | 9 | 18 | 18 | 165.5 | 1000000 | 99.54186683 |
| BD0063 | 56 | 179427 | 1575872 | 1667867 | 39.12 | 38.87 | 102417 | 102417 | 40627 | 39151 | 6 | 6 | 12 | 14 | 135 | 3100000 | 265.5672542 |
| BD0065 | 63 | 270362 | 1592158 | 1667867 | 39.14 | 38.87 | 64837 | 59572 | 37797 | 35104 | 7 | 8 | 15 | 17 | 134.5 | 700000 | 59.13357845 |
| BD0068 | 50 | 318469 | 1600238 | 1667867 | 38.9 | 38.87 | 65071 | 65071 | 43233 | 38907 | 7 | 7 | 14 | 15 | 146.5 | 1300000 | 119.0135467 |
| BD0069 | 29 | 233614 | 1611178 | 1667867 | 39.07 | 38.87 | 132757 | 130739 | 84716 | 55682 | 5 | 6 | 9 | 10 | 142 | 2400000 | 211.5222527 |
| BD0070 | 37 | 265525 | 1587934 | 1667867 | 38.93 | 38.87 | 108594 | 99791 | 55240 | 50174 | 4 | 5 | 10 | 11 | 163 | 1400000 | 143.7087436 |
| BD0072 | 83 | 265799 | 1609297 | 1667867 | 39.16 | 38.87 | 74030 | 66147 | 33667 | 28927 | 6 | 7 | 14 | 16 | 156.5 | 800000 | 77.79794531 |
| BD0073 | 45 | 284948 | 1644350 | 1667867 | 38.87 | 38.87 | 82315 | 80552 | 39428 | 37900 | 7 | 8 | 15 | 16 | 155 | 900000 | 84.83595342 |
| BD0076 | 37 | 173440 | 1556137 | 1667867 | 39.13 | 38.87 | 101535 | 101110 | 59033 | 51837 | 6 | 7 | 11 | 12 | 170.5 | 500000 | 54.78309429 |
| BD0077 | 1207 | 38084 | 2550386 | 1667867 | 38.91 | 38.87 | 3192 | 5073 | 1650 | 3302 | 204 | 95 | 486 | 197 | 150.5 | 2100000 | 123.922418 |
| BD0078 | 44 | 159562 | 1656774 | 1667867 | 38.73 | 38.87 | 101714 | 101714 | 68500 | 68500 | 7 | 7 | 12 | 12 | 159.5 | 1000000 | 96.27142869 |
| BD0080 | 36 | 226868 | 1584240 | 1667867 | 38.89 | 38.87 | 83498 | 80083 | 52801 | 51527 | 6 | 7 | 12 | 13 | 152.5 | 1000000 | 96.26066758 |
| BD0081 | 25 | 397278 | 1549815 | 1667867 | 39.21 | 38.87 | 151784 | 151784 | 95341 | 59392 | 4 | 4 | 7 | 8 | 177.5 | 400000 | 45.81191949 |
| BD0082 | 45 | 396258 | 1625016 | 1667867 | 38.84 | 38.87 | 78113 | 78113 | 37509 | 35328 | 6 | 6 | 13 | 14 | 169.5 | 700000 | 73.0146657 |
| BD0084 | 248 | 94631 | 1812299 | 1667867 | 38.9 | 38.87 | 44333 | 46110 | 18758 | 28445 | 17 | 15 | 31 | 26 | 158 | 400000 | 34.87283279 |
| BD0086 | 95 | 125954 | 1602447 | 1667867 | 39.13 | 38.87 | 54137 | 54137 | 32375 | 29711 | 11 | 11 | 20 | 22 | 171 | 800000 | 85.36943812 |
| BD0090 | 37 | 268919 | 1558874 | 1667867 | 39.08 | 38.87 | 62495 | 59764 | 45685 | 38822 | 6 | 7 | 14 | 16 | 154.5 | 600000 | 59.46599918 |
| BD0091 | 44 | 289033 | 1553891 | 1667867 | 39.2 | 38.87 | 89548 | 84519 | 60352 | 53316 | 5 | 6 | 11 | 12 | 134.5 | 800000 | 69.24552623 |
| BD0094 | 44 | 251058 | 1568553 | 1667867 | 39.11 | 38.87 | 80485 | 60511 | 40524 | 36697 | 5 | 6 | 13 | 15 | 132.5 | 900000 | 76.02548336 |
| BD0095 | 54 | 162333 | 1615143 | 1667867 | 38.92 | 38.87 | 67728 | 67728 | 34088 | 33767 | 9 | 9 | 17 | 19 | 132 | 800000 | 65.38120773 |
| BD0096 | 44 | 205460 | 1617192 | 1667867 | 38.86 | 38.87 | 61657 | 59936 | 41213 | 40367 | 6 | 7 | 14 | 15 | 132.5 | 2000000 | 163.8642783 |
| BD0097 | 39 | 228537 | 1607872 | 1667867 | 38.93 | 38.87 | 91671 | 91671 | 60780 | 59352 | 6 | 6 | 11 | 12 | 156 | 1100000 | 106.7249134 |
| BD0101 | 62 | 118065 | 1653177 | 1667867 | 38.75 | 38.87 | 71724 | 71724 | 32945 | 32061 | 9 | 9 | 18 | 19 | 146.5 | 900000 | 79.75552527 |
| BD0103 | 58 | 215984 | 1562157 | 1667867 | 39.01 | 38.87 | 57755 | 55406 | 33474 | 32732 | 7 | 8 | 17 | 19 | 128.5 | 600000 | 49.35483437 |
| BD0104 | 50 | 174307 | 1661402 | 1667867 | 38.84 | 38.87 | 67039 | 67039 | 38558 | 38558 | 8 | 8 | 16 | 16 | 163 | 500000 | 49.05495479 |
| BD0107 | 48 | 232055 | 1611901 | 1667867 | 39.02 | 38.87 | 143382 | 143382 | 37920 | 36651 | 5 | 5 | 11 | 12 | 156.5 | 800000 | 77.67226399 |
| BD0109 | 32 | 195706 | 1567439 | 1667867 | 39.09 | 38.87 | 90655 | 90655 | 52735 | 42206 | 7 | 7 | 12 | 14 | 175.5 | 700000 | 78.37625579 |
| BD0110 | 41 | 229297 | 1534575 | 1667867 | 39.11 | 38.87 | 100247 | 85957 | 46498 | 41193 | 6 | 7 | 12 | 14 | 172.5 | 800000 | 89.92717853 |
| BD0111 | 36 | 243971 | 1640136 | 1667867 | 38.79 | 38.87 | 97688 | 97688 | 46083 | 46083 | 6 | 6 | 13 | 13 | 162 | 1500000 | 148.1584454 |
| BD0112 | 33 | 230721 | 1620336 | 1667867 | 38.94 | 38.87 | 95201 | 88686 | 72044 | 71435 | 6 | 7 | 11 | 12 | 173 | 400000 | 42.70719159 |
| BD0114 | 59 | 176197 | 1593684 | 1667867 | 39.04 | 38.87 | 56612 | 56612 | 37731 | 37042 | 8 | 8 | 16 | 17 | 173.5 | 500000 | 54.43362674 |
| BD0115 | 51 | 237184 | 1634451 | 1667867 | 38.94 | 38.87 | 51283 | 50032 | 34909 | 34150 | 7 | 8 | 17 | 18 | 188.5 | 200000 | 23.06584902 |
| BD0119 | 84 | 176678 | 1631015 | 1667867 | 38.94 | 38.87 | 53399 | 53399 | 29145 | 28780 | 9 | 9 | 19 | 20 | 70.5 | 4000000 | 172.8984712 |
| BD0120 | 68 | 230571 | 1642419 | 1667867 | 39.02 | 38.87 | 69162 | 69162 | 34395 | 33019 | 8 | 8 | 16 | 17 | 154 | 500000 | 46.88206846 |
| BD0127 | 65 | 171585 | 1610787 | 1667867 | 38.91 | 38.87 | 59456 | 59456 | 32452 | 30234 | 10 | 10 | 19 | 20 | 170.5 | 600000 | 63.50932805 |
| BD0130 | 43 | 305084 | 1661114 | 1667867 | 38.82 | 38.87 | 126734 | 126734 | 68204 | 68204 | 5 | 5 | 10 | 10 | 154.5 | 600000 | 55.80592301 |
| BD0133 | 86 | 247775 | 1722293 | 1667867 | 38.64 | 38.87 | 52523 | 52523 | 34004 | 34733 | 9 | 9 | 20 | 19 | 157 | 900000 | 82.04178964 |

**Supplementary Table 2. List of genes evaluated and the present-absent status**

| **Cluster Names** | **Genes** | **Present** | **Absent** | **Percentage of presence** | **Non-Synonimous SNP** |
| --- | --- | --- | --- | --- | --- |
| cluster | Ars | 56 | 0 | 100% | 203 |
| cluster_1 | Toxin-Antitoxin_HP0968 | 54 | 2 | 96% | 69 |
| cluster_2 | *aibA* | 56 | 0 | 100% | 101 |
| cluster_3 | *alpB* | 56 | 0 | 100% | 207 |
| cluster_4 | *alpA* | 56 | 0 | 100% | 165 |
| cluster_5 | *flaA* | 56 | 0 | 100% | 12 |
| cluster_6 | *cheP* | 54 | 2 | 96% | 270 |
| cluster_7 | *murF* | 56 | 0 | 100% | 148 |
| cluster_8 | *futB* | 54 | 2 | 96% | 197 |
| cluster_9 | *amiA* | 56 | 0 | 100% | 167 |
| cluster_10 | *csd4* | 56 | 0 | 100% | 111 |
| cluster_11 | *tolB* | 56 | 0 | 100% | 59 |
| cluster_12 | *cagE* | 41 | 15 | 73% | 92 |
| cluster_13 | *rpoN* | 56 | 0 | 100% | 100 |
| cluster_14 | *gluP* | 56 | 0 | 100% | 78 |
| cluster_15 | *csd3* | 56 | 0 | 100% | 88 |
| cluster_16 | *cgt* | 56 | 0 | 100% | 66 |
| cluster_17 | *flgR* | 56 | 0 | 100% | 65 |
| cluster_18 | *csd6* | 56 | 0 | 100% | 49 |
| cluster_19 | *pseB* | 56 | 0 | 100% | 70 |
| cluster_20 | *csd1* | 56 | 0 | 100% | 75 |
| cluster_21 | *csd2* | 56 | 0 | 100% | 62 |
| cluster_22 | *cheY* | 56 | 0 | 100% | 57 |
| cluster_23 | *cheA* | 56 | 0 | 100% | 272 |
| cluster_24 | *motA* | 56 | 0 | 100% | 61 |
| cluster_25 | *motB* | 56 | 0 | 100% | 47 |
| cluster_26 | *fliA* | 56 | 0 | 100% | 36 |
| cluster_27 | *aibB* | 55 | 1 | 98% | 92 |
| cluster_28 | *arsR* | 56 | 0 | 100% | 46 |
| cluster_29 | *cagD* | 41 | 15 | 73% | 51 |
| cluster_30 | *csd5* | 50 | 6 | 89% | 136 |
| cluster_31 | *duf386* | 56 | 0 | 100% | 82 |
| cluster_32 | *cheW* | 56 | 0 | 100% | 22 |
| cluster_33 | *luxS* | 55 | 1 | 98% | 33 |
| cluster_34 | *spOT* | 56 | 0 | 100% | 126 |
| cluster_35 | *fur* | 56 | 0 | 100% | 12 |
| cluster_36 | *nikR* | 56 | 0 | 100% | 20 |
| cluster_38 | *napA* | 51 | 5 | 91% | 68 |
| cluster_39 | *ccmA* | 55 | 1 | 98% | 17 |
| cluster_40 | *cheY* | 56 | 0 | 100% | 1 |
| cluster_41 | *vapD* | 31 | 25 | 55% | 25 |
| cluster_42 | *homD* | 56 | 0 | 100% | 173 |
| cluster_43 | *flhA* | 56 | 0 | 100% | 46 |
| cluster_44 | *homB* | 54 | 2 | 96% | 279 |
| cluster_45 | *flgE* | 56 | 0 | 100% | 60 |
| cluster_46 | *tlpB* | 53 | 3 | 95% | 142 |

**Supplementary Table S3**. **Genes mutation of the Validation dataset (n=20)**

| Strain.ID | group | Biofilm.OD | *alpB* G160S | *alpB* A223V | *alpB* N156K | *gluP* T85S | *cgt* V34A | *csd5* P43S | *csd5* V110A | *csd5* M125I | *murF* V250I |
| --- | --- | --- | --- | --- | --- | --- | --- | --- | --- | --- | --- |
| JAY21 | 0 | 0.185 | S | A | N | S | V | S | A | I | V |
| KPG33 | 1 | 0.564 | G | V | K | T | A | P | V | M | I |
| MKS56 | 0 | 0.324 | K | A | N | S | V | S | T | I | V |
| SBY137 | 0 | 0.381 | S | A | N | T | V | P | V | M | V |
| MN3 | 0 | 0.302 | S | A | N | S | V | S | A | I | V |
| JAY1 | 1 | 0.511 | R | A | N | S | V | S | T | I | V |
| MER20 | 1 | 0.401 | S | V | N | S | V | Q | A | I | V |
| MN11 | 0 | 0.324 | S | A | N | S | V | S | T | I | V |
| KPG28 | 1 | 0.953 | S | A | N | T | A | S | V | M | V |
| NIAS56 | 1 | 0.624 | S | A | N | S | V | S | T | I | V |
| Manado29 | 0 | 0.380 | S | A | N | - | V | S | V | I | V |
| IND7 | 0 | 0.244 | S | A | N | T | V | S | V | M | V |
| MER21 | 0 | 0.309 | S | D | N | S | V | Q | V | I | V |
| SBY283 | 0 | 0.241 | G | A | N | S | V | S | V | I | V |
| JAY6 | 0 | 0.149 | S | V | N | S | V | Q | A | I | V |
| SMS30 | 1 | 0.414 | G | I | N | S | V | S | T | I | V |
| KPG64 | 1 | 0.440 | S | A | N | S | V | S | I | I | V |
| PTN63 | 0 | 0.377 | S | A | N | S | V | S | T | I | I |
| SMS22 | 0 | 0.339 | G | A | N | S | V | S | A | I | V |
| NIAS37 | 0 | 0.297 | S | A | N | S | V | S | T | I | V |
| **High+mutant** | | | 71.4 | 42.9 | 14.3 | 71.4 | 28.6 | 85.7 | 71.4 | 71.4 | 14.3 |
| **Low+mutant** | | | 84.6 | 15.4 | 0.0 | 84.6 | 0.0 | 92.3 | 61.5 | 84.6 | 14.3 |


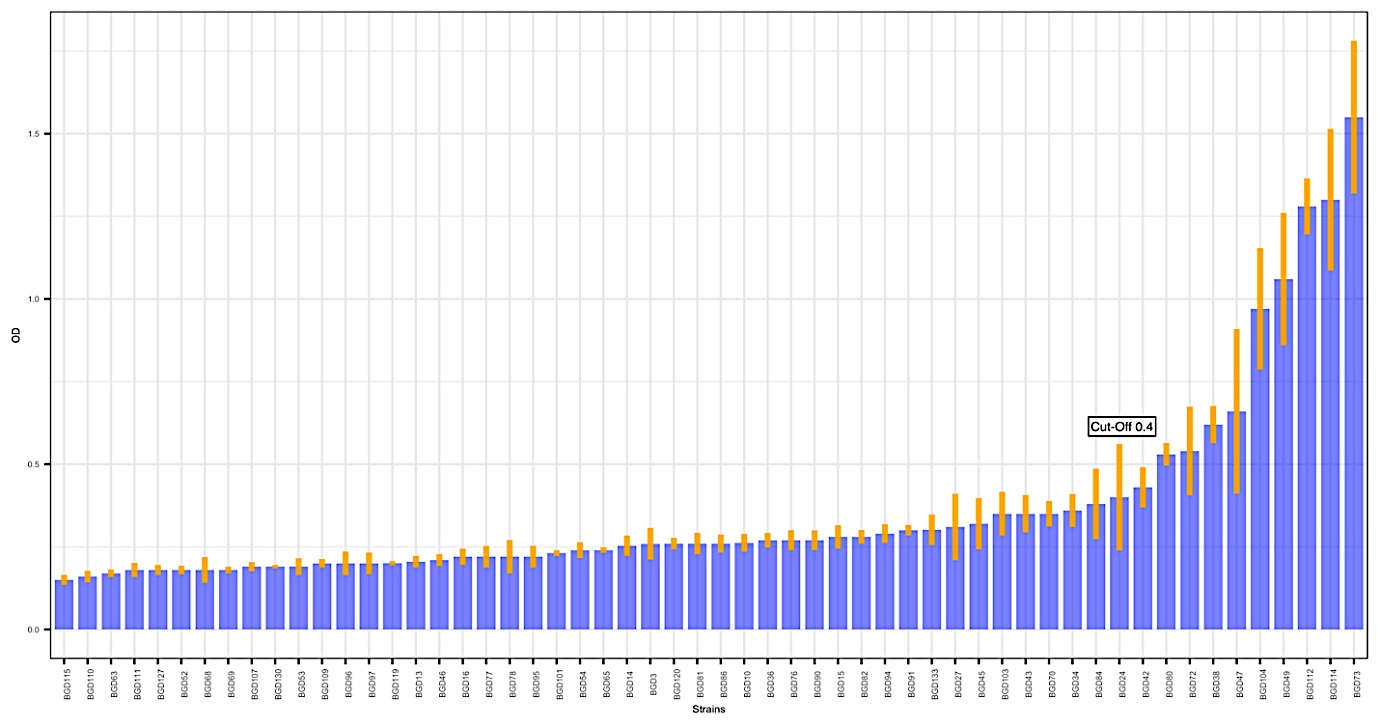


**Supplementary Figure S1.** Distribution of biofilm formation in strains from 56 patients from Bangladesh. The x-axis represented the strains and y-axis was the optical density of biofilm formation after crystal violet staining (reduced with the OD of the blank plates without samples). The cutoff (0.4) divided the strains into High and Low biofilm former. Lower: The biofilm formation relative to the growth OD at Day 4.


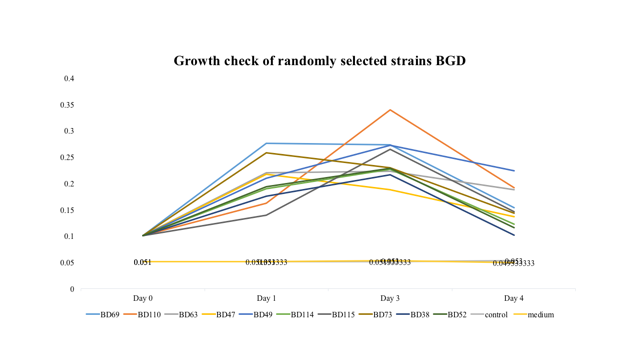


**Supplementary Figure S2a.** Bacterial growth curve of Bangladesh strain from Day 0 until the Day 4 as indicated by Optical Density (OD) measurement. The y- axis is the Optical Density, x-axis is the days of measurement and each strains represented by different strains. This showed that the bacterial growth does not significantly different between the strains.


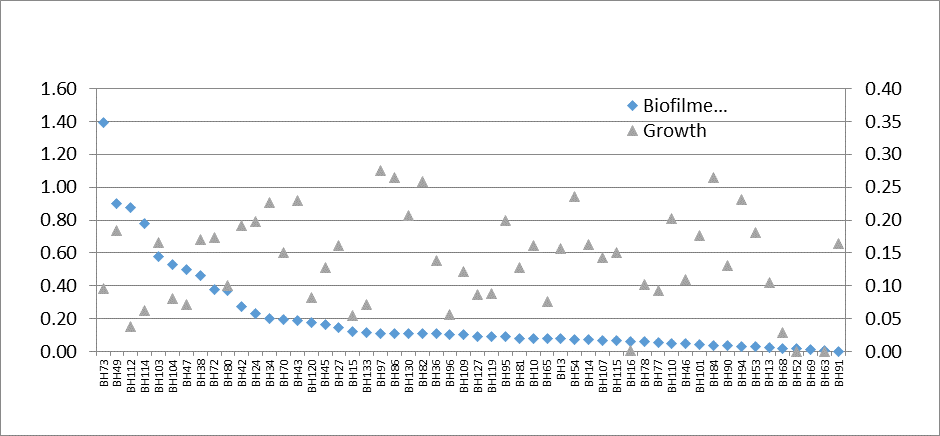


**Supplementary Figure S2b**. The graph of Day 4 bacterial growth (gray triangle) and biofilm formation (blue square) indicated by optical density. The left y-axis represented the biofilm optical density (0- 1.60 OD) and right y- axis. represented the bacterial growth OD (0-0.40 OD).

**Supplementary Figure S3.** The expression of *alpB gene*  that are normalized with the *ppa*  housekeeping gene. The *y-axis*  is the absolute quantity of the 120ng total RNA and *x-axis* represented isolates` name. The green bar represented the high biofilm former isolates (BGD114, BGD112, BGD104) while the orange bar are the low biofilm former isolates (BGD96 and BGD109).


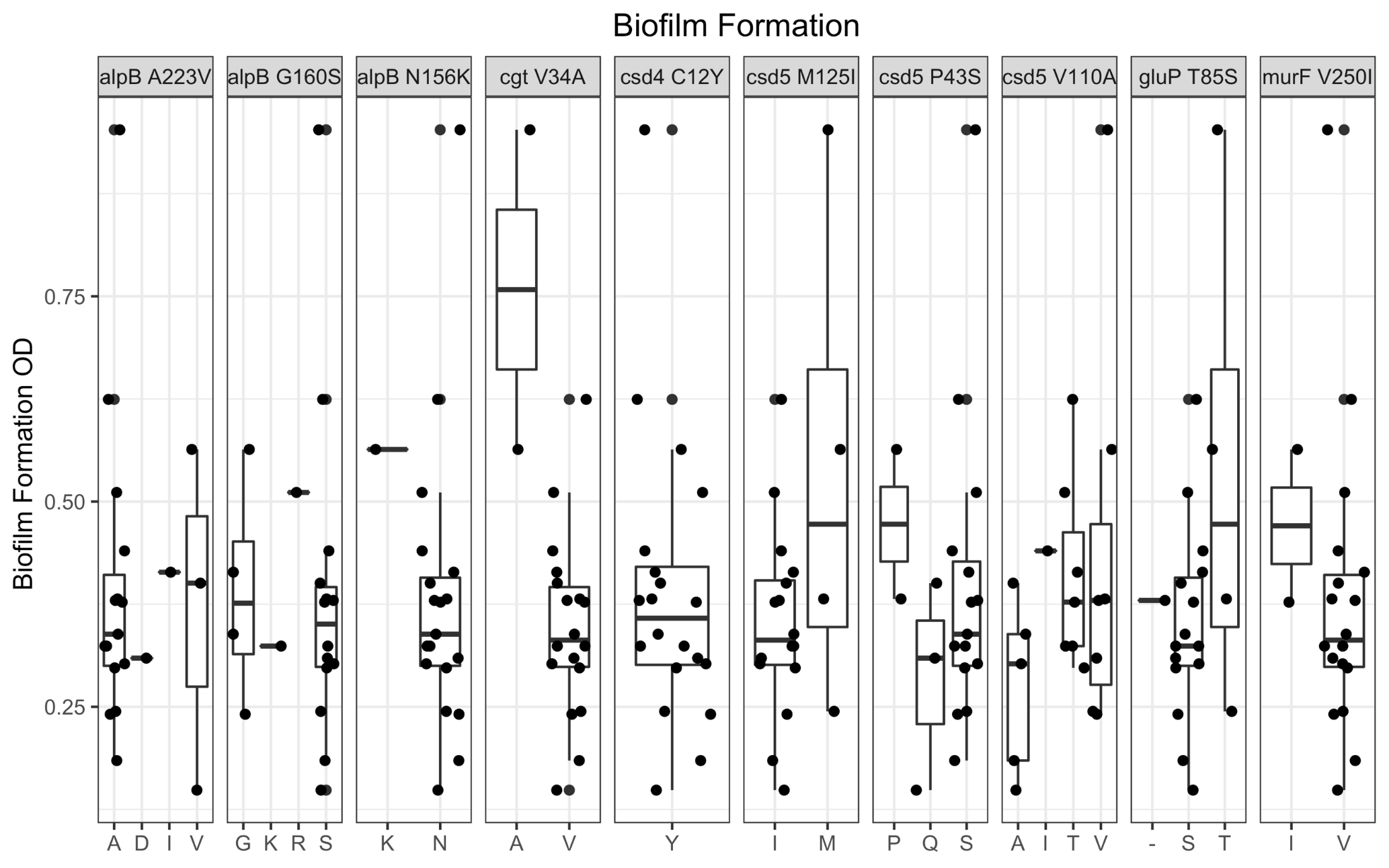


**Supplementary Figure S4.** The presence of mutation of *alpB, cgt, csd4, csd5, gluP*  and *murF*  in the new dataset genome (n=20)*.*  The *Y- axis*  represent the biofilm formation. Among those genes, the allele present in each locus were shown.
